# Amyloid-β: Structure, Function, and Pathophysiological Significance in Neurodegenerative Diseases

**DOI:** 10.3390/ijms231810275

**Published:** 2022-09-07

**Authors:** Satoshi Saito, Kenjiro Ono, Masashi Tanaka

**Affiliations:** 1 Department of Neurology, National Cerebral and Cardiovascular Center, 6-1 Kishibe-Shimmachi, Osaka 564-8565, Japan; 2 Department of Neurology, Kanazawa University Graduate School of Medical Sciences, 13-1 Takara-machi, Kanazawa 920-8640, Japan; 3 Department of Physical Therapy, Health Science University, 7187 Kodachi, Fujikawaguchiko-machi, Minamitsuru-gun, Yamanashi 401-0380, Japan

The rate of dementia continues to increase worldwide; however, there currently exist no therapeutic strategies for this condition. Although several effective predictive markers (e.g., plasma amyloid-β [Aβ]_42_/Aβ_40_ ratios) for Alzheimer’s disease (AD) have been identified, further studies are needed to identify more sophisticated and less expensive predictive markers for dementia.

Recent extensive studies revealed the pathological implications of Aβ in the development and progression of dementia. Aβ monomers are prone to aggregation, which forms toxic Aβ oligomers that cause neuronal and vascular injuries. Aβ is also involved in other cytotoxic mediators, such as reactive oxygen species (ROS) and neuroinflammation, thereby suggesting complex pathogenesis and leading to the development and progression of neurodegenerative diseases, including AD and cerebral amyloid angiopathy. Accordingly, there exists an urgent need to elucidate the molecular mechanisms underlying Aβ aggregation processes, ROS generation, and neuroinflammation to identify effective therapeutic targets and identify and develop drugs/bioactive molecules with preventive and/or therapeutic potentials for Aβ-related neurodegenerative diseases.

As such, this Special Issue includes 15 original manuscripts, a case report, a commentary, and review articles that contribute to the aforementioned aim and provide novel insights into the mechanisms underlying the pathogenesis of Aβ-related neurodegenerative diseases.

We have included several interesting papers that address the mechanisms of action of Aβ aggregation and oligomerization. Banerjee et al. demonstrated that the interaction between Aβ and cellular membrane triggered the on-membrane self-assembly of Aβ, thereby promoting the oligomer formation of Aβ [1]. Furthermore, Aβ aggregates bound to the cellular membrane acted as seeds for further aggregation, resulting in cell permeability and damage and subsequently inducing cell lysis, as shown by Ruiz-Arias et al. using a mouse neuroblastoma cell line [2]. These findings highlight the significance of Aβ dynamics on the cellular membrane in forming oligomers and exhibiting cytotoxicity in physiological conditions. Regarding familial AD, He et al. investigated the effects of several types of Aβ mutations on the secondary structure and subsequent aggregation and showed novel roles of these mutations in AD pathogenesis [3]. In a case report, Shimada et al. characterized a recently identified Osaka mutation in the gene amyloid precursor protein (APP) [4]. They found that patients with dementia carrying this mutation had a high tau burden and subtle Aβ accumulation in the cerebral cortex and cerebellum, thereby suggesting tau accumulation and neurodegeneration through toxic Aβ oligomers without senile plaque formation [4].

Neuroinflammation has been closely implicated in the pathogenesis of neurodegenerative diseases, which involves a shift in microglial phenotypes from homeostasis to a proinflammatory state. By analyzing the brains of patients with AD, Walker et al. showed that the expression levels of purinergic adenosine diphosphate/triphosphate receptor P2RY12 on microglia defines the boundary between the proinflammatory area, consisting of microglia interacting with and/or adjacent to Aβ plaques, and the nonaffected area away from the Aβ plaques [5]. These findings suggest that P2RY12 carries a novel pathological significance in the proinflammatory axis of microglia around Aβ plaques in AD brains [5].

A commentary paper by Friedland et al. provided a unique point of view on the pathogenesis of neurodegenerative diseases. Notably, they summarized the potential roles of gut microbiota-derived amyloid proteins in the aggregation of neuronal proteins, such as Aβ, and in neuroinflammation [6].

This Special Issue also includes papers that addressed the preventive and/or therapeutic potentials of chemical compounds or drugs for Aβ-related neurodegenerative diseases. Murakami et al., who focused on 10-Me-Aplog-1, a new protein kinase C activator, demonstrated its inhibitory effects on the intracellular formation of toxic Aβ oligomers in rat primary cerebral cortex cells [7]. In a mouse model of cerebral amyloid angiopathy, Yakushiji et al. showed that the administration of low-dose phosphodiesterase III inhibitor cilostazol improved vascular deposition of Aβ, potentially by facilitating perivascular drainage of Aβ [8]. These findings would promote research aimed at developing novel drugs and identifying repositioning drugs for Aβ-related neurodegenerative diseases.

We have also included excellent review articles in this Special Issue, which provide updates on unique topics concerning neurodegenerative diseases.

Tomiyama and Shimada, who summarized the characteristics of Osaka mutation in the APP gene, argued that the loss-of-function in APP and gain-of-function in Aβ were caused by this mutation and were implicated in the pathogenesis of AD [9]. Regarding Aβ aggregation, Watanabe-Nakayama et al. comprehensively reviewed the usefulness of high-speed atomic force microscopy to visualize the structural dynamics in the aggregation process of Aβ, which would lead to novel insights into the mechanisms underlying Aβ aggregation [10].

Regarding preventive and/or therapeutic potentials for Aβ-related neurodegenerative diseases, Ono and Tsuji reported the significance of Aβ protofibrils as a therapeutic target in AD while describing the pathological implications of Aβ protofibrils in AD [11]. Furthermore, Tadokoro et al. focused on the roles of ROS in the Aβ cascade and pathogenesis of AD. Notably, they summarized the recent findings regarding the pathological relationship between ROS and AD and described the beneficial effects of antioxidative supplements on AD pathogenesis [12]. Apart from antioxidants, several natural medicines that could beneficially affect memory decline in AD through pleiotropic mechanisms of action are emerging, as reviewed by Kuboyama et al. [13]. Conversely, there are natural compounds that would need careful consideration during administration. In line with this, Kobayashi et al. showed that some naturally occurring polyphenols suppressed Aβ aggregation and were expected to have protective effects against AD; however, some of them pose the potential risk of oxidative damage due to pro-oxidant properties [14]. As another therapeutic approach for AD, Iqbal et al. described the therapeutic potential of anti-infectious drugs based on the possibility that Aβ plaque formation is the innate immune response against microorganisms in AD brains [15].

We earnestly believe that the excellent papers included in this Special Issue improve our understanding of the pathogenesis of Aβ-related neurodegenerative diseases and help develop effective preventive and therapeutic strategies for such diseases.

## Data Availability

Not applicable.
